# Phase 1 study of intravenous administration of the chimeric adenovirus enadenotucirev in patients undergoing primary tumor resection

**DOI:** 10.1186/s40425-017-0277-7

**Published:** 2017-09-19

**Authors:** Rocio Garcia-Carbonero, Ramon Salazar, Ignacio Duran, Ignacio Osman-Garcia, Luis Paz-Ares, Juan M. Bozada, Valentina Boni, Christine Blanc, Len Seymour, John Beadle, Simon Alvis, Brian Champion, Emiliano Calvo, Kerry Fisher

**Affiliations:** 1 Instituto de Biomedicina de Sevilla, IBiS/Hospital Universitario Virgen del Rocío/CSIC/Universidad de Sevilla, Seville, Spain; 2 Medical Oncology Department, Catalan Institute of Oncology, IDIBELL, University of Barcelona, Barcelona, Spain; 3 Unidad de Urología-Oncológica, UGC de Urología y Nefrología, Instituto de Biomedicina de Sevilla, IBiS/Hospital Universitario Virgen del Rocío/CSIC/Universidadde Sevilla, Seville, Spain; 4 START Madrid, Centro Integral Oncológico Clara Campal, Hospital Madrid Norte Sanchinarro, Madrid, Spain; 5 PsiOxus Therapeutics Limited, Milton Park, Abingdon, UK; 6 Department of Oncology, Oxford University, Oxford, UK

## Abstract

**Background:**

Enadenotucirev (formerly ColoAd1) is a tumor-selective chimeric adenovirus with demonstrated preclinical activity. This phase 1 Mechanism of Action study assessed intravenous (IV) delivery of enadenotucirev in patients with resectable colorectal cancer (CRC), non-small-cell lung cancer (NSCLC), urothelial cell cancer (UCC), and renal cell cancer (RCC) with a comparator intratumoral (IT) dosed CRC patient cohort.

**Methods:**

Seventeen patients scheduled for primary tumor resection were enrolled. IT injection of enadenotucirev (CRC only) was administered as a single dose (≤ 3 × 10^11^ viral particles [vp]) on day 1, followed by resection during days 8–15. IV infusion of enadenotucirev was administered by three separate doses (1 × 10^12^ vp) on days 1, 3, and 5, followed by resection during days 8–15 (CRC) or days 10–25 (NSCLC, UCC, and RCC). Enadenotucirev activity was measured using immunohistochemical staining of nuclear viral hexon and quantitative polymerase chain reaction for viral genomic DNA.

**Results:**

Delivery of enadenotucirev was observed in most tumor samples following IV infusion, with little or no demonstrable activity in normal tissue. This virus delivery (by both IV and IT dosing) was accompanied by high local CD8^+^ cell infiltration in 80% of tested tumor samples, suggesting a potential enadenotucirev-driven immune response. Both methods of enadenotucirev delivery were well tolerated, with no treatment-associated serious adverse events.

**Conclusions:**

This study provides key delivery and feasibility data to support the use of IV infusion of enadenotucirev, or therapeutic transgene-bearing derivatives of it, in clinical trials across a range of epithelial tumors, including the ongoing combination study of enadenotucirev with the checkpoint inhibitor nivolumab. It also provides insights into the potential immune-stimulating properties of enadenotucirev.

**Trial registration:**

This MOA study was a phase 1, multicenter, non-randomized, open-label study to investigate the administration of enadenotucirev in a preoperative setting (ClinicalTrials.gov: NCT02053220).

**Electronic supplementary material:**

The online version of this article (doi:10.1186/s40425-017-0277-7) contains supplementary material, which is available to authorized users.

## Background

Oncolytic viruses hold great promise for the effective treatment of cancer because of their unique cell-killing mechanisms, tumor-selective replication, amplification of the initial therapy dose through replication in vivo, and potential to provoke an anticancer immune response [1, 2]. Although a variety of genetically modified viruses have been developed as oncolytic agents [1, 3, 4], our incomplete understanding of both tumor and virus biology can limit optimal design of oncolytic viruses [5, 6]. Therefore, a more effective approach may be to avoid rational design of viruses altogether in favor of directed evolution (i.e. genetic diversification followed by phenotypic selection) [5, 6]. Enadenotucirev (formerly known as ColoAd1), a novel group B Ad11p/Ad3 chimeric adenovirus, is the first oncolytic virus to be successfully developed using this approach [7]. Enadenotucirev is more potent than its parental strains and other wild-type or genetically engineered adenoviruses that it has been compared with to date, and, in terms of both virus replication and killing of tumor cell lines and primary tissues, has demonstrated greater tumor selectivity, particularly against epithelial carcinomas [7–12].

The primary oncolytic action of enadenotucirev is through a direct non-apoptotic, pro-inflammatory cell-killing mechanism, similar to oncosis or ischemic cell death [10]. As non-apoptotic lytic pathways of cell death can be highly immunogenic [13], oncolytic viruses may also create a pro-inflammatory environment and stimulate an anticancer immune response [14]. In addition, a recent study has shown that enadenotucirev directly participates in innate immune activation via the CD46 receptor on dendritic cells [15].

Systemic delivery through intravenous (IV) infusion is a major goal in the delivery of oncolytic viruses, so that the agent is able to access both the primary tumor and metastatic lesions [1, 16]. To date, talimogene laherparepvec (an attenuated herpes simplex virus type-1 produced in Vero cells by recombinant DNA technology) is the only oncolytic virus therapy to be licensed by the European Medicines Agency and the US Food and Drug Administration – for the local treatment of unresectable melanoma [3, 4, 17]. However, talimogene laherparepvec is delivered by intratumoral (IT) injection, restricting its use to accessible tumors and requiring specialist skills, particularly for non-superficial tumors.

Historically, many studies have failed to demonstrate appreciable antitumor activity when oncolytic viruses are delivered via IV infusion [18, 19], probably stemming from a combination of neutralizing antibodies, antiviral cytokine response, complement-mediated inactivation, uptake by Kupffer cells, and erythrocyte sequestration [14, 20–22]. Novel design strategies, such as antigenic masking [23] and immunosuppression [24], are being explored as strategies to resolve these difficulties.

Enadenotucirev was prioritized for clinical development and delivery by IV infusion on the basis of preclinical evidence of its stability in human whole blood [25] and the low prevalence of neutralizing antibodies against group B adenoviruses (including Ad11p, the exclusive component of the outer coat of enadenotucirev) in the general population [26, 27].

This phase 1 Mechanism Of Action (MOA) study was conducted to assess the pattern of viral delivery and viral expression, anti-tumor immune response, and safety of enadenotucirev delivered by IV infusion or IT injection in patients with colorectal cancer (CRC) and other epithelial tumors. The data demonstrate that enadenotucirev can access and replicate within tumor tissue, with successful delivery regardless of the route of administration. The data also provide some evidence supporting the stimulation of an induced immune response by enadenotucirev. Both modes of delivery are shown to be feasible and well tolerated.

## Methods

### Study design and treatment regimens

**Fig. 1:**
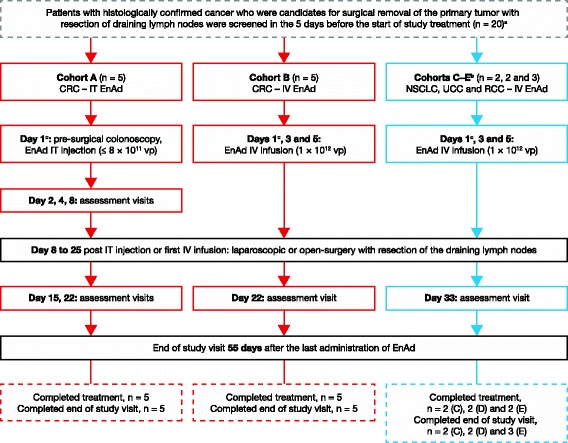
Study design and patient disposition. a Two patients were excluded at screening because of inadequate renal function, and one because of bowel obstruction. b Cohorts C–E initiated after completion of cohort A and B comparison phase. c Enadenotucirev (EnAd) administration days also counted as assessment visits (i.e. cohort A: day 1; cohort B: days 1, 3, and 5)

The study was initially planned in two cohorts of patients with CRC, to investigate the route of enadenotucirev administration: IT injection (cohort A) and IV infusion (cohort B; Fig. 1). It was then extended to investigate IV infusion of enadenotucirev in patients with non-small-cell lung cancer (NSCLC, cohort C), urothelial cell cancer (UCC, cohort D), and renal cell cancer (RCC, cohort E). The main eligibility criteria included age 18 years or older, and histologically confirmed early stage CRC, NSCLC, UCC, or RCC, and a primary tumor diameter of at least 3 cm, scheduled for tumor resection. For patients with CRC in cohort A, the tumor had to be accessible by colonoscopy in order to allow IT injection of enadenotucirev. Patients were not eligible if they had rectal tumors or obstructive tumors of the intestine or urinary tract, another primary malignancy (except for non-melanoma skin cancer or cervical cancer in situ) in the previous 3 years, known central nervous system metastases, or any known condition necessitating tumor resection within 8–10 days of enadenotucirev administration. The treatment period comprised a single cycle of treatment, surgery, and follow-up visits, lasting 22 days in patients with CRC and 33 days in patients with other tumor types. The end-of-study visit was 55 days after the last administration of enadenotucirev, or 28 days after surgery, whichever occurred later.

Patients with CRC in cohort A received a single IT injection of enadenotucirev at the time of presurgical colonoscopy (study day 1). A variable volume of the diluted virus (1 × 10^11^ viral particles [vp]/mL) was administered using repeated needle insertions; the dose was based on the available surface area of the tumor, up to a planned maximum dose of 8 × 10^11^ vp (8 mL); the actual doses administered were 0.6–3.0 × 10^11^ vp. This was followed by laparoscopic or open surgery during days 8–15. The dose for the IT injection was determined from preclinical data, aiming to achieve measurable transgene expression while minimizing any spill - over into the bloodstream.

Patients with CRC in cohort B received three separate IV infusions of diluted virus (1 × 10^12^ vp) on days 1, 3, and 5, each given over 5 min, followed by laparoscopic or open surgery during days 8–15. Patients with NSCLC, UCC, or RCC (cohorts C–E) also received three separate doses of diluted virus (1 × 10^12^ vp) by IV infusion on days 1, 3, and 5, but followed by laparoscopic or open surgery during days 10–25 (in one patient, surgery was delayed until day 52 due to surgeon decision). The dose of enadenotucirev for IV infusion was determined to be safe and tolerable from early data of the preliminary phase 1 Evolve study (NCT02028442; manuscript in preparation).

### Histopathology and immunohistochemistry

The tissue samples obtained during surgery were staged by the local pathologist and then prepared for specific analyses. Up to three tumor blocks, one visually normal tissue block from the tumor margin, and one draining lymph node block (when available) were fixed in 10% neutral buffered formalin or 4% neutral buffered formaldehyde for 18–24 h and then embedded in paraffin. Some larger tissue blocks were further fragmented into 2–4 pieces each and re-embedded to aid sectioning and to enable evaluation of more regions of the tumor. A set of 21 sections was prepared from each tissue block for histopathologic assessment by a GCP-compliant laboratory (Targos Molecular Pathology GmbH, Kassel, Germany), in order to determine the extent of delivery and spread of enadenotucirev, and to detect any evidence of an immune response. If sufficient tumor had been resected and the patient had provided separate consent, additional tissue samples were frozen at −20 °C for exploratory analyses (detection of viral DNA using quantitative polymerase chain reaction [qPCR]).

Immunohistochemistry (IHC) was performed on sections 6, 9, 12, 15, 18, and 21 to detect enadenotucirev hexon – the major adenovirus capsid protein. Hexon protein is expressed late during infection, only after viral genome replication has occurred, and is transported to the nucleus for final virus assembly, where it usually forms honeycomb-like intranuclear inclusion bodies. Nuclear detection of inclusion bodies in tumor tissue block sections therefore indicates that the virus has undergone all the major steps of replication. The staining procedure used a pan-hexon monoclonal antibody (1E11, Abcam, Cambridge, UK) and was validated according to the ICH Q2 (R1) guidelines. Staining was detected using a Ventana Ultra View Detection Kit (Ventana Medical Systems Inc., Tucson, AZ, USA). Staining of enadenotucirev in each section was assessed by a board-certified pathologist and the proportion of tumor cells with positive staining (0–100%) determined.

Sections 1 and 2 were initially stained for enadenotucirev hexon protein or an immunoglobulin G (IgG) isotype control antibody, section 3 was used for hematoxylin and eosin staining for histopathologic subtype assessment, and the remaining sections were used for other inflammatory and virus receptor stains, including cluster of differentiation (CD) 8, CD46, CD11b, CD25, CD57, and desmoglein 2 (DSG2). Blocks were also stained for the DNA mismatch repair proteins MLH1 and MSH2. From the majority of patients with CRC, further sections were subsequently prepared from two or three blocks of resected tumor tissues and stained for the immune markers CD4, forkhead box P3 (FoxP3), and programmed cell death 1 (PD-1) at Pathology Diagnostics Ltd. (Cambridge, UK). Scoring of CD8 cell infiltration into tumor cell nests (as opposed to tumor stroma) was assessed according to the method of Naito et al. [28]. For each available stained section scanned image, a 1 mm^2^ area with the most abundant distribution of CD8 cells was selected, the number of CD8 cells located within tumor cell nests was counted by two independent scientists, using ImageJ software, and the average of the two counts was taken for assessment of the degree of CD8 infiltration according to the following scoring criteria: 0 = No CD8 cells; 1–19 = Low; 20–49 = Moderate; ≥ 50 = High.

### Measurement of enadenotucirev in blood by qPCR

The concentration of enadenotucirev in blood was measured using qPCR to assess the pharmacokinetics of enadenotucirev immediately following IT injection and to confirm virus concentrations following IV infusion. A validated qPCR assay was used in a GCP-compliant laboratory (BioOutsource Ltd., Glasgow, UK). The qPCR primers used were:

1. forward primer: ATCRCATGTCTAGACTTCGACRCCAG
2. reverse primer: TGCTGGGTGATAACTATGGGGT
3. probe: 6ʹFAM-ATCTGTGGAGTTCATCGCTCTCTTACG-3ʹTAMRA.
4. The qPCR product is 2859 base pairs across the E2B region of enadenotucirev.

Whole-blood samples were frozen immediately after collection and stored at –20 °C until analysis. Blood samples were spiked with bovine adenovirus to act as a DNA extraction control; if bovine adenovirus recovery was less than 50%, the sample extraction was repeated. Each sample was tested in triplicate and the mean for each sample calculated.

### Detection of enadenotucirev in tumor samples by qPCR

Exploratory qPCR was used to detect enadenotucirev genomic DNA, in order to support the IHC hexon staining data. Samples were semi-thawed and then cut into small pieces using a sterile scalpel; each piece was then weighed, fully lysed, and the DNA extracted. Samples were analyzed by qPCR, as described above for detecting the concentration of enadenotucirev in blood.

### Viral shedding detected by qPCR (IT injection only)

Measurements of enadenotucirev shedding were performed for cohort A only. Urine samples were obtained by clean-catch urine collection, saliva samples were collected using buccal swabs, and fecal samples were collected using rectal swabs. These samples were collected before IT injection of enadenotucirev and then on days 1, 2, 4, 8, 15, and 22, and at the end-of-study visit. The samples were analyzed by qPCR, as described above.

### Antibody response

For cohort A, serum was sampled before and after IT injection of enadenotucirev on day 1, on days 8, 15, and 22, and at the end-of-study visit. For cohorts B–E, serum was sampled before and after IV infusion on day 1, on day 22 (cohort B) or before surgery on the day of surgery (cohorts C–E), and at the end-of-study visit.

Serum samples (prediluted 1:100 to avoid serum inhibition) were analyzed using an enzyme-linked immunosorbent assay with electrochemiluminescence detection (Meso Scale Diagnostics, Rockville, MD, USA). Briefly, assay plates were coated with enadenotucirev for 1 h at a concentration of 3.6 × 10^10^ vp/mL. After washing the plates, serially titrated patient serum samples were added and incubated for 1 h, followed by washing and addition of the biotinylated anti-human IgG Fc detection antibody (BioLegend, Cat. No. 409308) at 1 μg/mL. This detection antibody cross-reacts with rabbit IgG, allowing the use of a rabbit anti-enadenotucirev antiserum as a positive control in the assays. After an hour, plates were washed and sulfo-tag labelled streptavidin (0.2 μg/mL) added for a further hour and then processed for signal detection with an MSD plate reader. Data were expressed as the reciprocal dilution generating a statistically determined positive signal above background. Titers below 1/11 dilution were considered as negative.

### Cytokine response (IV infusion only)

Concentrations of interleukin (IL)-2, IL-4, IL-6, IL-10, IL-12, interferon (IFN)γ, monocyte chemoattractant protein (MCP)-1, and tumor necrosis factor (TNF) in serum were assessed for cohorts B–E (i.e. after IV infusion). Serum samples were collected before IV infusion of enadenotucirev, 6–8 h after infusion on days 1, 3 and 5, and before surgery on the day of surgery. Serum samples were analyzed using a Luminex bead-based multiplex assay (R&D Systems, Abingdon, UK) by ACM Global Laboratories (York, UK).

## Safety assessments

A complete medical history was taken and a physical examination performed at screening. Limited symptom-directed physical examinations were performed at all subsequent visits. Safety assessments were conducted at each study visit. These included recording of adverse events (AEs), from the time of informed consent until the end-of-study visit, and laboratory safety tests (hematology, coagulation profile, chemistry, and urinalysis). Vital signs (systolic and diastolic blood pressure, heart rate, oral temperature, and respiratory rate), Eastern Cooperative Oncology Group performance status [29], body weight, and height (for calculation of body mass index) were also recorded.

## Results

### Patient population

Twenty patients were screened for study eligibility (Fig. 1), of whom 17 were enrolled in the study and received treatment. None of the patients had received any tumor-related treatment before enrolment. Patients received study treatment between 1 October 2013 and 6 February 2015. Sixteen patients completed study treatment; one patient (in cohort E) discontinued study treatment because of a stoma site infection before the day 5 IV infusion of enadenotucirev. All 17 patients who received at least one dose of enadenotucirev underwent surgical resection of their primary tumor and safety evaluations, and all attended the end-of-study visit.

Ten patients with CRC entered the first stage of the study; five were treated with enadenotucirev administered by IT injection (cohort A) and five with enadenotucirev administered by IV infusion (cohort B). Seven additional patients, two with NSCLC (cohort C), two with UCC (cohort D), and three with RCC (cohort E), entered the second stage of the study and received enadenotucirev administered by IV infusion.

There were no notable differences in baseline characteristics between cohorts A and B (Additional file 2: Table S1).

### Feasibility and safety profile

Administration of enadenotucirev by IT injection and IV infusion was feasible and generally well tolerated (Table 1). Exposure to enadenotucirev was lower with IT injection (single administration of 0.6–3 × 10^11^ vp) than with IV infusion (1 × 10^12^ vp administered over 5 min on days 1, 3, and 5 in 11 patients, and on days 1 and 3 in one patient [the day 5 dose was missed]), reflecting the direct administration into the tumor.

**Table 1:** Adverse events in the safety population following IT injection or IV infusion

	CRC (IT injection, *n* = 5)	CRC, NSCLC, UCC, RCC (IV infusion, *n* = 12)
Any AE, *n* (%)^a^	5 (100.0)	12 (100.0)^b^
Treatment-related AE, *n* (%)	0 (0)	10 (83.3)
Asthenia^a^	0	4
Chills	0	3
Neutropenia	0	3
Pyrexia	0	3
Any SAE, *n* (%)	1 (20.0)	3 (25.0)
Abdominal abscess	1	0
Enteritis	0	1
Subcutaneous emphysema	0	1
Wound dehiscence	0	1
Treatment-related SAE	0 (0.0)	0 (0.0)
Any grade 3 or 4 AEs, *n* (%)	2 (40.0)	3 (25.0)
Abdominal abscess	1	0
Anemia	2	0
Asthenia	0	1
Enteritis	0	1
Gastrointestinal hemorrhage	1	0
Hypertension	0	1
Hypocalcemia	1	0
Hypokalemia	1	0
Oliguria	0	1
Wound dehiscence	0	1
Treatment-related grade 3 or 4 AEs, *n* (%)	0 (0.0)	0 (0.0)
AE leading to study discontinuation, *n* (%)	0 (0.0)	1 (8.3)
Stoma site infection	0	1

^a^The most commonly reported AEs following IV infusion were pyrexia (58.3% of patients), asthenia (51.7% of patients), abdominal pain (33.3% of patients), and neutropenia (33.3% of patients)

^b^Specific AEs are listed by occurrence rather than number of patients reporting them (i.e. a patient can have more than one concomitant AE)

All patients had at least one AE (any grade), and overall, more AEs were reported following IV infusion than after IT injection. Anemia was the only event reported in more than one patient following IT injection but was not considered to be related to treatment. Treatment-related AEs were reported by 83.3% of patients following IV infusion; all were considered mild or moderate in severity (grade 1 or 2). The most commonly reported treatment-related AEs following IV infusion were asthenia (33.3% of patients), neutropenia, chills, and pyrexia (each 25.0% of patients). There were no treatment-related deaths, treatment-related serious AEs (SAEs), or treatment-related AEs that led to discontinuation. No treatment-related AEs were reported following IT injection. Grade 3 laboratory abnormalities were reported in three patients after administration of enadenotucirev; one of these, low potassium on day 15 after IT administration of enadenotucirev, was considered to be of clinical significance.

### Enadenotucirev hexon staining

**Fig. 2:**
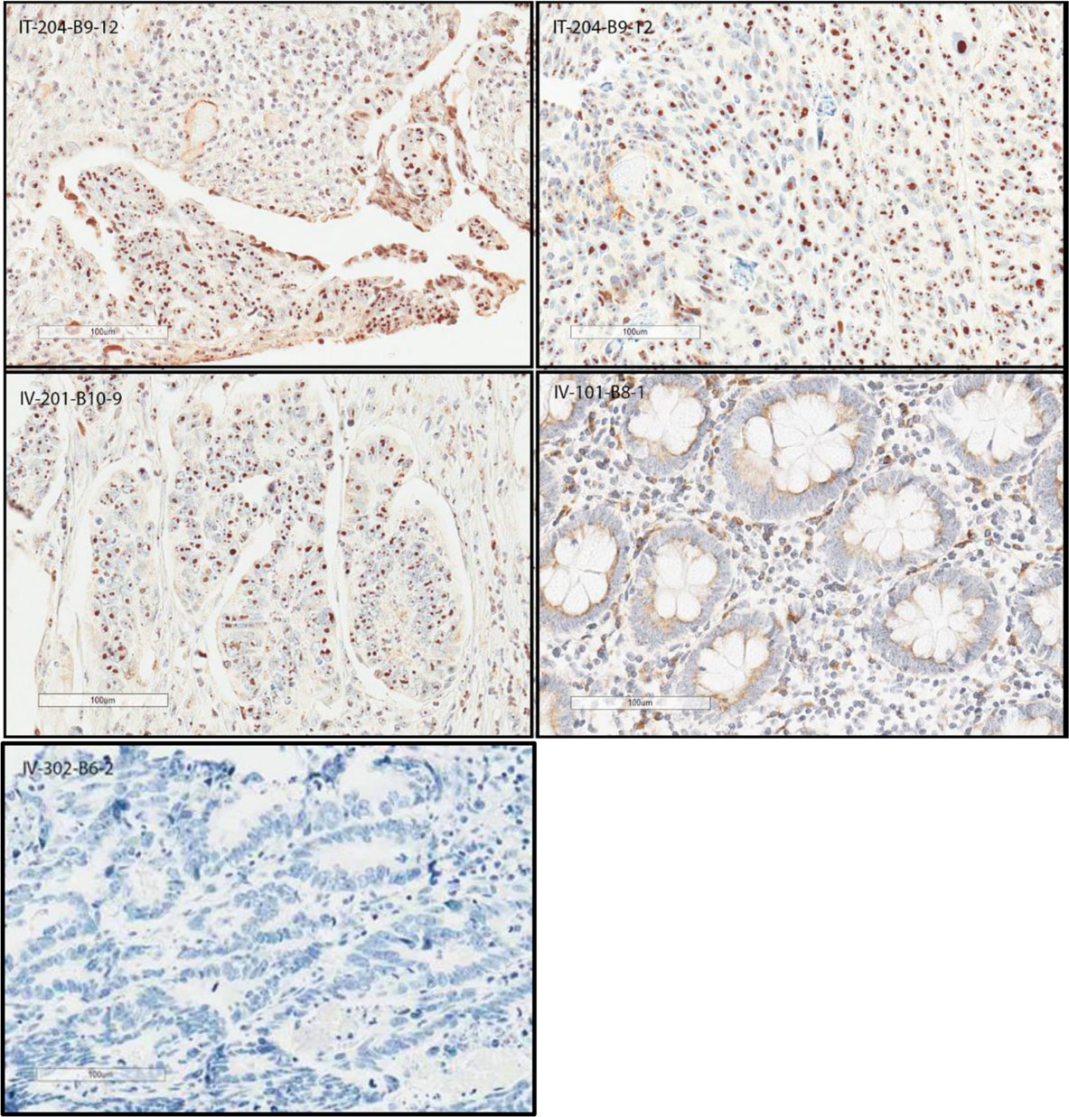
Nuclear staining of enadenotucirev in samples from selected CRC patients (IT injection versus IV infusion). 1. Image IT-204-B9-12 (top left), tumor section from a patient with CRC treated with enadenotucirev given by IT injection (cohort A): strong brown nuclear hexon staining of tumor cells is visible in the bottom part of the tissue section. 2. Image IT-204-B9-12 (top right), a different region of the same tumor section in 1: nuclear hexon staining of tumor cells is visible throughout this area of the tissue section. 3. Image IV-201-B10-9 (middle left), tumor section from a patient with CRC treated with enadenotucirev given by IV infusion (cohort B) but whose resection was delayed until day 52: nuclear hexon staining of tumor cells (but not stroma) is still visible in this tissue. 4. Image IV-101-B8-1 (middle right), non-tumor (presumed normal) section from a patient with CRC treated with enadenotucirev given by IV infusion (cohort B): little or no nuclear hexon staining is visible throughout the tissue section. 5. Image IV-302-B6-2 (bottom left), isotype staining control

IHC assessments for enadenotucirev were performed on 446 samples, including tumor material and normal tissue from the 10 patients with CRC. Lymph nodes, inflammatory tissue, and tubular adenoma were also available for analysis from some patients. Clear evidence of enadenotucirev in the tumors (punctate brown staining of virus hexon protein in the nuclei) was found in all tumor samples; examples are provided in Fig. 2. Within these tumor sections, nuclear staining was observed in the epithelially derived tumor cells, whereas cells in the stroma and visually normal colon epithelial tissues generally did not stain (see Additional file 2: Table S2 and Additional file 1: Figure S1 for more details of non-tumor staining). Resection was delayed to day 52 in one patient (IV0201); enadenotucirev was still detectable in the tumor sample from this patient. Since the adenovirus hexon protein is dependent on the virus major late promoter, and is only produced subsequent to viral DNA synthesis [30], the staining data also provided evidence that virus replication had also initiated by the time samples were taken.

The distribution of enadenotucirev nuclear staining through the tumor sections is summarized in Table 2. After IV infusion, positive tumor cell staining was observed in all 224 sections stained and in the majority of sections (125 of 147) stained after IT injection. This difference is likely to be due to the focal deposition of the virus following IT injection.

**Table 2:** Nuclear staining of enadenotucirev in patients with CRC (IT injection vs IV infusion)

Cohort	Patient	Total number of sections	Nuclear staining (%)						
			0	> 0 ≤ 20	> 20 ≤ 40	> 40 ≤ 60	> 60 ≤ 80	> 80 ≤ 100	No tumor
A (IT injection)	IT0201	42	0	31	8	3	0	0	0
	IT0203	14	0	0	0	2	11	1	0
	IT0204	42	1	0	0	0	4	30	7
	IT0301	21	7	14	0	0	0	0	0
	IT0302	28	14	11	2	1	0	0	0
	Total, n (%)	147 (100.0)	22 (15.0)	56 (38.1)	10 (6.8)	6 (4.1)	15 (10.2)	31 (21.1)	7 (4.8)
B (IV infusion)	IV0101	49	0	38	8	3	0	0	0
	IV0201^a^	21	0	0	3	0	11	7	0
	IV0301	70	0	25	11	13	6	8	7
	IV0302^b^	63	0	21	9	9	4	6	14
	IV0303	21	0	11	7	3	0	0	0
	Total^b^, n (%)	224 (100.0)	0 (0.0)	95 (42.4)	38 (17.0)	28 (12.5)	21 (9.4)	21 (9.4)	21 (9.4)

^a^Patient did not have surgery until 51 days after the first IV infusion of enadenotucirev

^b^Number corrected from data source

### Immune-cell infiltration in tumors

**Fig. 3:**
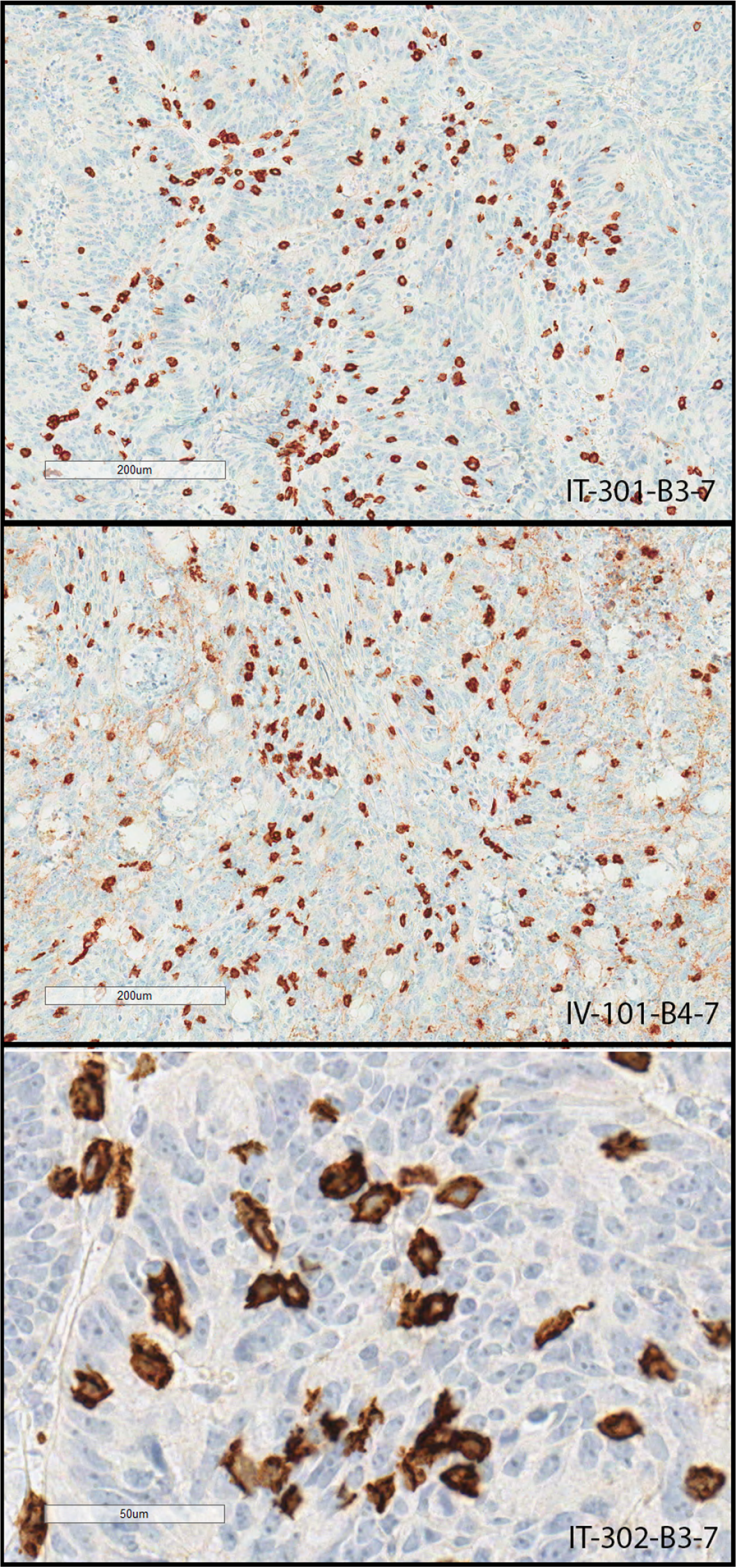
CD8 staining in samples from CRC patients (IT injection versus IV infusion). 1. Image IT-301-B3-7 (top panel), tumor section from a patient with CRC treated with enadenotucirev given by IT injection (cohort A): brown CD8 staining (× 10 magnification) can be clearly seen infiltrating the tumor cells. 2. Image IV-101-B4-7 (middle panel), tumor section from a patient with CRC treated with enadenotucirev given by IV infusion (cohort B): brown CD8 staining (× 10 magnification) infiltrating the tumor cells can be clearly seen. 3. Image IT-302-B3-7 (bottom panel), tumor section from a patient with CRC treated with enadenotucirev given by IT injection (cohort A): higher magnification (× 40) showing closer detail of CD8-stained cells, which appear pleomorphic, a hallmark of activated CD8^+^ T cells

Eight of the 10 samples from patients with CRC showed high levels of CD8^+^ cell infiltration within the tumor cell nests (Table 3) and in the stromal tissue (representative images are shown in Fig. 3). Tumor samples IT0203 and IV0201 showed only low levels of tumor-associated CD8+ cells. No suitable baseline tumor biopsy material was available for analysis in parallel with the post-dose sections from these patients.

**Table 3:** Level of CD8 T-cell infiltration in tumor cell nests

Patient	CD8 cells in tumor cell nests	Infiltration level
IT-201	209	High
IT-203	18	Low
IT-204	206	High
IT-301	388	High
IT-302	169	High
IV-101	365	High
IV-201	4	Low
IV-301	116	High
IV-302	188	High
IV-303	154	High

^a^CD8 cell numbers were counted in a 1 mm^2^ area of tumor for two different tissue blocks per patient, as described in Methods, with the average of the two scores used here, with the exception of IT-204 and IV-201 where only one CD8 stained section was available

Although a direct comparison of CD8 cell numbers in tumor tissues before and after treatment was not possible, CD8^+^ T cells are generally considered to be rare in CRC tumors and, if seen at higher levels, are usually associated with a high level of microsatellite instability (MSI) [31]. However, none of the tumor samples were considered to have high levels of MSI (i.e. MLH1 and MSH2 expression was normal in all tumor blocks analyzed; Additional file 2: Table S3). Therefore one hypothesis for the presence of CD8^+^ cells would be that their entry was part of a localized immune response, potentially driven by enadenotucirev virus infection within the tumor. Using higher magnification, many of the CD8^+^ cells that infiltrated the tumor cell nests appeared to have a pleomorphic phenotype. The loss of a rounded phenotype is a hallmark of T-cell activation and provides further observational evidence for CD8^+^ T-cell activation [32].

To explore the nature of the immune cell infiltrates further, another immunostaining was carried out. PD-1 is a known co-marker with CD8 for tumor-infiltrating activated cytotoxic T cells [33]. As with CD8 staining, PD-1^+^ cells were detected within the tumor cell nests and in the stromal tissue (Additional file 1: Figure S2). By contrast, both CD4 and FoxP3 staining were primarily restricted to the stromal regions of tumor sections taken from patients with CRC regardless of whether they received enadenotucirev by IT injection or IV infusion (Additional file 1: Figure S2). CD4^+^ T-cells expressing FoxP3 are indicative of regulatory T cells (T_regs_) which are thought to promote tumor progression by suppressing antitumor immunity in the tumor microenvironment [32–34]. The compartmental co-localization of CD4^+^ and FoxP3^+^ cells suggests that a significant subset of the CD4^+^ cells in the stroma may have a regulatory T-cell function, although activated effector human T cells can also express the FoxP3 marker.

Of the other inflammatory cell markers, expression of CD11b (myeloid lineage marker) and CD25 (activated and regulatory T cells) was also seen primarily in stromal tissues, although the staining was generally quite weak and variable, making it difficult to draw any conclusions. CD57+ cells (a marker of natural killer cells) were rarely seen.

Lastly, we observed that all tumor samples from patients with CRC stained strongly with the group B adenovirus receptors CD46 and DSG2 (data not shown).

### Detection of enadenotucirev DNA in tumors

**Fig. 4:**
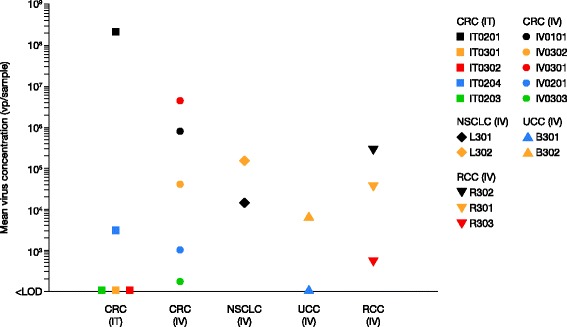
Enadenotucirev DNA detection in samples of epithelial tumors by qPCR (all cohorts). The exploratory qPCR analysis in patients with CRC and other tumor types provides supporting evidence demonstrating that enadenotucirev can be delivered to several different carcinoma types by IV infusion. Individual patient data are shown with corresponding identification in the key

As an exploratory alternative to staining of enadenotucirev hexons, qPCR was used to detect enadenotucirev DNA after enadenotucirev administration to patients (Fig. 4). Enadenotucirev DNA was detected after IV administration of enadenotucirev in tumor samples from 11 of 12 patients (five CRC, one UCC, three RCC, two NSCLC) and in two of five tumor samples after IT administration. The difference in the rate of detection of enadenotucirev DNA is probably due to the focal delivery by IT injection, which can lead to some tumor tissue sections being taken from uninjected areas (see also negative staining of some sections in Table 2).

### Detection of enadenotucirev DNA in blood

Enadenotucirev DNA levels in blood samples following IT injection in patients with CRC (cohort A) were below the quantification limit of the qPCR assay (data not shown). No enadenotucirev DNA was detected in blood samples before the first IV infusion of enadenotucirev on day 1 (cohorts B–E; Additional file 1: Figure S3) and a cumulative effect of dosing on enadenotucirev DNA level was not observed. The mean concentration of enadenotucirev in the blood was similar following IV infusions on days 1, 3, and 5. Results were also similar in samples from patients with different tumor types.

Patients with CRC had additional samples taken 6–8 h after each IV infusion of enadenotucirev. On days 3 and 5, there was an approximately 80-fold decrease in virus concentration over the 8 h following enadenotucirev infusion, which is consistent with a circulating virus half-life of approximately 20 min.

### Detection of enadenotucirev DNA in urine, saliva, and fecal samples (IT injection only)

Enadenotucirev DNA was not detected at significant levels by qPCR in any urine or saliva samples at any study time point (Additional file 2: Table S4). Enadenotucirev DNA was detected in fecal samples from all patients who received enadenotucirev by IT injection and was in the quantifiable range of the assay in two patients (IT0201 and IT0204).

### Enadenotucirev immunogenicity

The anti-enadenotucirev serum IgG titer before enadenotucirev administration was undetectable or low (below 1/11 negative threshold) in all blood samples from all patients. After administration of enadenotucirev by IT injection (cohort A), the mean anti-enadenotucirev titer remained low at all time points (Additional file 1: Figure S4). After IV infusion of enadenotucirev (cohorts B**–**E), the mean anti-enadenotucirev titer was elevated and peaked at day 22 in cohort B (1/79) and pre-surgery in cohorts C–E (cohort C, 1/149; cohort D, 1/174; cohort E, 1/167). Notably, antibody levels remained low (< 1/100) in two-thirds of the patients who received enadenotucirev by IV infusion. Antibody titers remained detectable in all IV infusion cohorts until study end. This study did not test whether these antibodies were neutralizing.

### Cytokine response (IV infusion only)

There was a trend for an increase in the concentrations of IL-6, IL-10, and MCP-1 at 6–8 h after IV infusion of enadenotucirev in the five patients with CRC in cohort B (data not shown) but, generally, the concentration had returned to the pre-infusion level at the next sample (48 h after dosing). No other cytokine (IL-2, IL-4, IL-12, IFNγ or TNF) showed any appreciable changes over time during or after IV administration of enadenotucirev (data not shown). Changes in cytokine responses were not evaluated in other patients because of sampling errors.

## Discussion

This study was designed to assess viral delivery, viral expression, and inflammatory infiltrates within tumor tissues when enadenotucirev is administered by either IT injection or IV infusion to patients with CRC, and by IV infusion to patients with several different epithelial tumor types.

Enadenotucirev administration, whether by IT injection or IV infusion, was feasible and generally well tolerated. There was no evidence that enadenotucirev resulted in any grade 3 or 4 AEs, SAEs, or AEs leading to study discontinuation. Treatment-related AEs were more frequent after IV infusion than after IT injection, but were mild and were anticipated on the basis of preliminary data from the EVOLVE study (manuscript in preparation) [35]. The lower number of events reported after IT injection than IV infusion may be explained by the lower systemic virus exposure following a lower total administered dose (estimated 2–10% of the dose given by IV infusion).

Short-term changes in cytokine levels are a specific potential safety issue with oncolytic viruses. In previous studies using high doses of systemically administered viruses, the cytokine profile has correlated closely with signs of toxicity [36], such as the commonly observed ‘flu-like’ response, with cytokine levels peaking 6–48 h after infusion and resolving 3–6 days later. Our results demonstrate that systemic cytokine responses vary greatly between patients. At 6–8 h after IV infusion of enadenotucirev, there was a trend for an increase in the levels of some inflammatory cytokines (IL-6, IL-10, and MCP-1) but levels generally returned to pre-infusion levels 48 h after dosing. We therefore conclude that IV infusion of enadenotucirev at the doses administered in this study is not associated with any of the serious cytokine release-related events reported with other oncolytic viral therapies.

Extensive IHC analysis of samples from patients with CRC clearly showed effective delivery of enadenotucirev to the tumor, with widespread nuclear hexon staining (indicative of viral replication) and generally weak or negative staining in non-tumor tissue. Successful delivery of virus to the tumor tissues was confirmed by qPCR measurement of virus genomic DNA content. This selective replication of enadenotucirev in tumor cells is consistent with preclinical studies [7] and was achieved irrespective of the enadenotucirev administration method. IV infusion of enadenotucirev is therefore feasible and may offer other advantages because it has the potential to target disseminated tumor sites. Although some clinical studies have reported occasional positive staining for oncolytic virus delivery (in biopsies from 10 to 20% of patients) [37–40], to our knowledge, this is the first clinical trial to clearly demonstrate consistent delivery of an oncolytic virus to tumors with a favorable safety profile following IV administration.

Further studies are required to determine the significance of enadenotucirev hexon staining found in some normal tissue and lymph nodes. Where staining did occur in these cells, it was weak, there was no sign of pathology, and the cells looked healthy. The proximity of the normal samples to the excised tumor margin in this study is unknown. It is therefore impossible to rule out a direct influence of the tumor on these cells, such as the effects of released cytokines, changes in vascular permeability, or uptake of hexon proteins from viruses released locally from lysed tumor cells. Furthermore, some normal tissue close to a tumor can have precancerous changes [41], which could predispose cells to tumor development in the absence of morphologic changes [42] and therefore increase the possibility of their sensitization to enadenotucirev replication. The specific cell type that stained positive for hexon in lymph nodes was not established in this study.

Our study also provides indirect evidence supporting the hypothesis that enadenotucirev may have stimulated an immune response in the tumors of patients prior to their resection. Large numbers of CD8^+^ cells were seen in most tumor samples from the patients with CRC. In CRC, CD8^+^ T-cell infiltration within tumor cell nests is rare and is usually associated with MSI status [31]. All CRC tumor samples in our study were microsatellite stable; therefore, the high CD8^+^ cell numbers may represent an immune response to the enadenotucirev virus infection rather than being induced by tumor hypermutation in the context of MSI, although baseline samples were not analyzed for CD8^+^ cell infiltration. The CD8 staining in these tumor samples had the same localization pattern as that of PD-1 staining, suggesting the possibility that many of these cells were activated cytotoxic T cells (CTLs). A closer examination of their cellular morphology revealed that many of these cells were pleomorphic, consistent with a CTL activation phenotype. Whether these putative CTLs could recognize viral antigens from infected tumor cells and/or immunologically processed tumor antigens from lysed cells, or even whether they are pre-existing CTLs that were inactivated within the tumor microenvironment, is not known.

In contrast to CD8, CD4 staining was almost exclusively restricted to stromal regions. Furthermore, the FoxP3 staining pattern aligned with that of CD4, perhaps reflecting a CD4^+^/FoxP3^+^ T_reg_ subset. T_reg_ infiltration of tumor sites is a well-documented feature of many forms of cancer, their presence contributing to a highly immunosuppressive microenvironment that facilitates tumor survival [34, 43]. In our study, the localization of the T_reg_ phenotypic marker FoxP3 to non-tumor stromal regions, coupled with potentially activated CTL tumor infiltrates, indirectly provides further support for the hypothesis that enadenotucirev replication may be able to counterbalance localized T_reg_-mediated immunosuppression.

Tumor cells present other barriers to an effective anti-cancer immune response. For example, while activated CD8^+^/PD-1^+^ CTLs can infiltrate tumors, they are frequently thwarted in their anti-cancer role by tumor cell expression of PD-1 ligand 1 (PD-L1), directly stimulated by key CTL cytokines such as IFNγ, triggering a PD-1 dependent deactivation state known as ‘adaptive resistance’ [33]. Staining for PD-L1 was variable and inconclusive (data not shown). It is possible that the CD8^+^ cells we observed in the tumor samples still face this obstacle despite the physical exclusion of T_reg_ cells. Combining the immune-stimulating properties of oncolytic viruses with therapies designed to interfere with ligand recognition by PD-1 may be key to unlocking this deadlock [44–46], a hypothesis that will be tested in a phase 1 trial of enadenotucirev in combination with the PD-1 inhibitor nivolumab (NCT02636036, SPICE).

Less extensive IHC was performed on samples from patients with NSCLC, UCC, or RCC. Technical issues made it difficult to draw firm conclusions about virus delivery and activity in bladder, lung, and renal carcinomas using this analysis. The exploratory qPCR analysis of enadenotucirev genomic DNA in tumor tissue frozen at the time of resection did, however, indicate virus delivery to these tumors after IV infusion as well as to patients with CRC after administration by both the IV and IT routes.

The virus particle kinetics of enadenotucirev (in whole blood) administered by IV infusion suggest that repeated infusion has a negligible impact on the clearance of the virus. Enadenotucirev DNA was detectable following IV infusion, but no significant levels of enadenotucirev DNA were detectable in the bloodstream after IT injection. The mean concentration of DNA was similar after each dose administered by IV infusion, indicating that there was no cumulative effect, and the kinetics appeared to be similar in the different tumor types. Enadenotucirev DNA was detected in all fecal samples, although it was not possible to determine whether the genome was intact or partially degraded. This may reflect leakage of the virus from the site of IT injection in the colon tumor.

An intrinsic advantage of enadenotucirev is its potentially low immunogenicity, due to exclusive expression of group B (Ad11p) viral coat proteins [26, 27]. Therefore, it was unsurprising that all patients in the MOA study had negligible levels of pre-existing antibodies to enadenotucirev. Nevertheless, this is an important observation because enadenotucirev is unlikely to be neutralized by patient antibodies immediately after administration. Anti-enadenotucirev antibody titers were not raised in patients following IT injection, whereas IV infusion appears to elicit an antiviral immune response in some, but not all, patients over time. However, with a single cycle of treatment, as used in this study, antibodies do not appear to play an important role in limiting delivery by IV infusion.

## Conclusions

This study provides valuable early safety, targeting, kinetic, and immunological information to support the future clinical development of enadenotucirev for systemic administration by IV infusion, including the ongoing combination study of enadenotucirev with the checkpoint inhibitor nivolumab. We have shown that enadenotucirev can gain access to and replicate within different types of epithelial tumors, underlining its broad therapeutic potential. This study also sheds light on the pro-inflammatory nature of enadenotucirev.

## Additional files

Additional file 1: **Figure S1.** Example of selective staining of tumor tissue compared with normal tissue after IV infusion of enadenotucirev (patient IV0302). Figure shows selective staining of tumor tissue (right-hand side of margin) compared with normal epithelial cells (left-hand side). **Figure S2.** Representative CD8, CD4, PD-1, and FoxP3 staining (Patient IV0101). This series of IHC stains illustrates a general finding amongst patients treated with enadenotucirev: that PD-1 aligns with CD8, and FoxP3 aligns with CD4. **Figure S3.** Virus kinetics in patients with CRC (cohort B) during treatment with enadenotucirev (EnAd) administered by IV infusion. Each data point represents the mean value of all patients with CRC treated with enadenotucirev (IV infusion) over the 5-day treatment period (pre-treatment, immediately after infusion, and 6–8 h after infusion) and a single pre-surgery sample. Limit of assay quantification (LOQ) ~ 2 × 10^5^ vp. Similar data were observed for cohorts C–E (data not shown). **Figure S4.** Detection of anti-enadenotucirev (EnAd) antibodies in patients following treatment with enadenotucirev. All samples were diluted 1:100 prior to analysis (to avoid serum inhibition). The titer shown is not corrected for this pre-dilution. (DOCX 3462 kb)

Additional file 2: **Table S1.** Patient characteristics. **Table S2.** Nuclear staining of enadenotucirev in non-tumor tissue samples from patients with CRC (IT injection vs IV infusion). **Table S3.** Expression of the DNA mismatch repair proteins MLH1 and MSH2 in tumor samples from patients with CRC (IT injection and IV infusion). **Table S4.** Viral shedding in patients with CRC treated with enadenotucirev by IT injection (cohort A). (DOCX 80 kb)

## Abbreviations

AE: Adverse event
CD: Cluster of differentiation
CRC: Colorectal cancer
CTL: Cytotoxic T cell
DSG2: Desmoglein 2
FoxP3: Forkhead box P3
GCP: Good Clinical Practice
ICH: International Conference on Harmonisation
IFN: Interferon
IgG: Immunoglobulin G
IHC: Immunohistochemistry
IL: Interleukin
IT: Intratumoral
IV: Intravenous
MCP: Monocyte chemoattractant protein
MOA: Mechanism of action
MSI: Microsatellite instability
NSCLC: Non-small-cell lung cancer
PD-1: Programmed cell death 1
PD-L1: PD-1 ligand 1
qPCR: Quantitative polymerase chain reaction
RCC: Renal cell cancer
SAE: Serious adverse event
TNF: Tumor necrosis factor
T_reg_: Regulatory T cell
UCC: Urothelial cell cancer
vp: Viral particles
