# Supplementary material for: Phase 1 study of intravenous administration of the chimeric adenovirus enadenotucirev in patients undergoing primary tumor resection
Source: J Immunother Cancer. 2017 Sep 19;5:71. doi: 10.1186/s40425-017-0277-7 (PMC5604344; doi:10.1186/s40425-017-0277-7)
Supplement: Supplementary file 1 — Figure S1. Example of selective staining of tumor tissue compared with normal tissue after IV infusion of enadenotucirev (patient IV0302). Figure shows selective staining of tumor tissue (right-hand side of margin) compared with normal epithelial cells (left-hand side). Figure S2. Representative CD8, CD4, PD-1, and FoxP3 staining (Patient IV0101). This series of IHC stains illustrates a general finding amongst patients treated with enadenotucirev: that PD-1 aligns with CD8, and FoxP3 aligns with CD4. Figure S3. Virus kinetics in patients with CRC (cohort B) during treatment with enadenotucirev (EnAd) administered by IV infusion. Each data point represents the mean value of all patients with CRC treated with enadenotucirev (IV infusion) over the 5-day treatment period (pre-treatment, immediately after infusion, and 6–8 h after infusion) and a single pre-surgery sample. Limit of assay quantification (LOQ) ~ 2 × 10^5^ vp. Similar data were observed for cohorts C–E (data not shown). Figure S4. Detection of anti-enadenotucirev (EnAd) antibodies in patients following treatment with enadenotucirev. All samples were diluted 1:100 prior to analysis (to avoid serum inhibition). The titer shown is not corrected for this pre-dilution. (DOCX 3462 kb) [file 40425_2017_277_MOESM1_ESM.docx]

# Additional File 1

Additional Figure 1.

Example of selective staining of tumor tissue compared with normal tissue after IV infusion of enadenotucirev (patient IV0302).


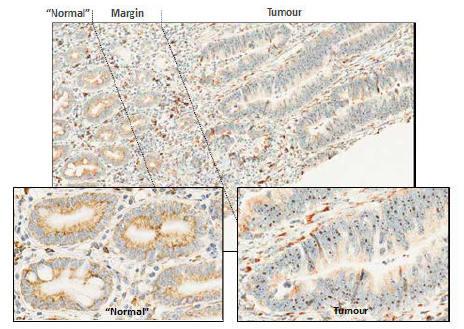


Figure shows selective staining of tumor tissue (right-hand side of margin) compared with normal epithelial cells (left-hand side).

Additional Figure 2.

Representative CD8, CD4, PD-1, and FoxP3 staining (Patient IV0101).


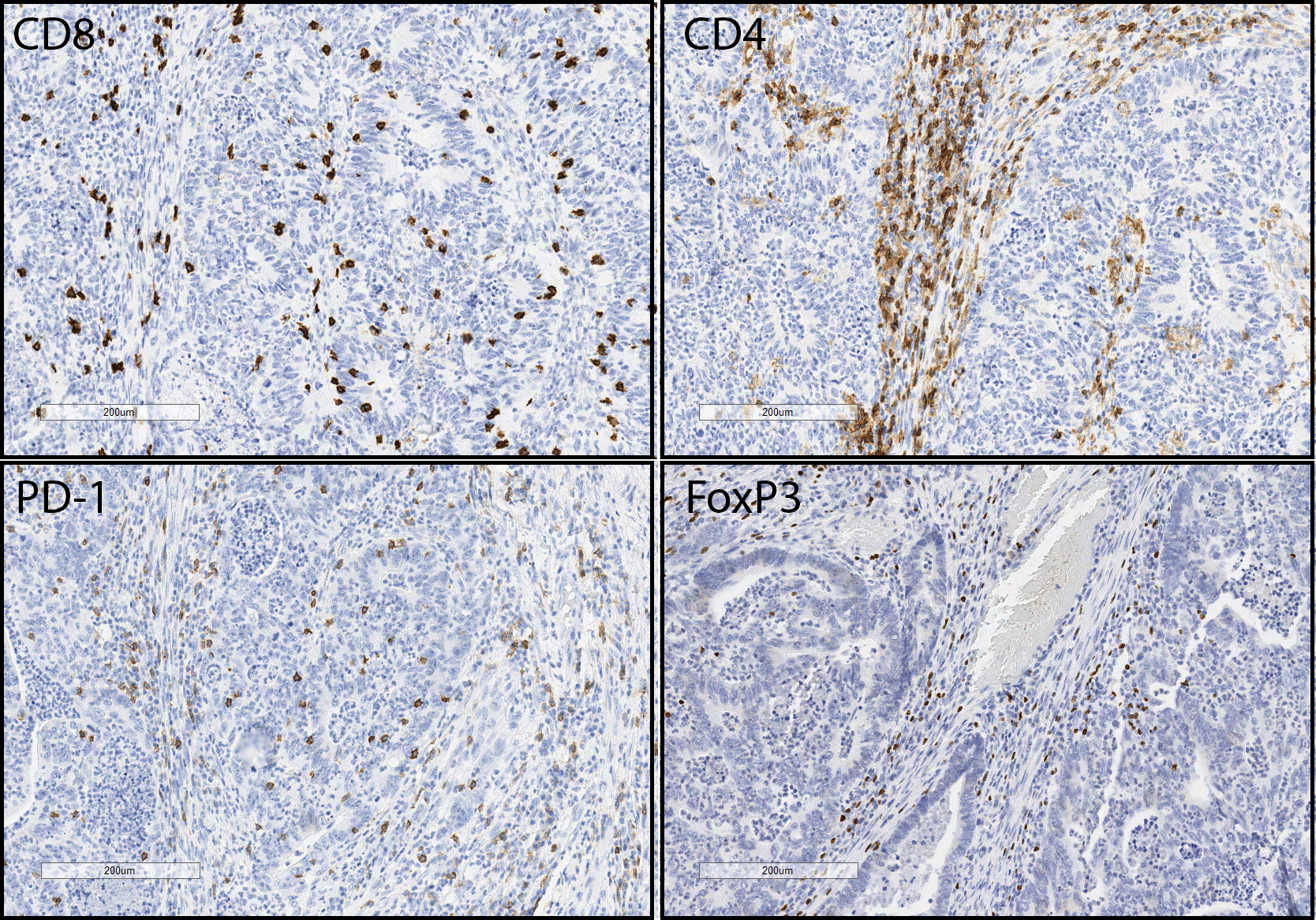


This series of IHC stains illustrate a general finding amongst patients treated with enadenotucirev: that PD-1 aligns with CD8, and FoxP3 aligns with CD4.

Additional Figure 3.

Virus kinetics in patients with CRC (cohort B) during treatment with enadenotucirev (EnAd) administered by IV infusion.


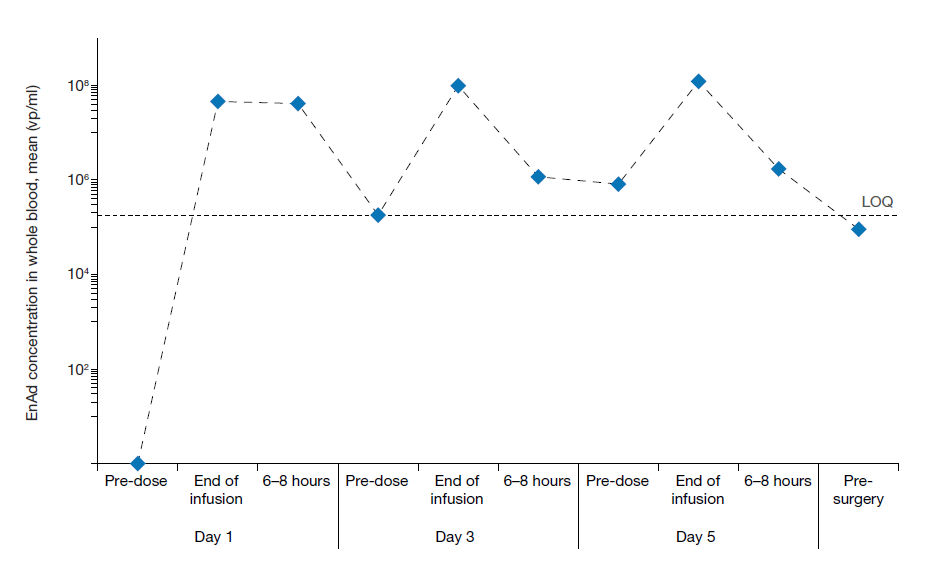


Each data point represents the mean value of all patients with CRC treated with enadenotucirev (IV infusion) over the 5-day treatment period (pre-treatment, immediately after infusion, and 6–8 hours after infusion) and a single pre-surgery sample. Limit of assay quantification (LOQ) ~ 2 × 10^5^ vp. Similar data were observed for cohorts C–E (data not shown).

**Additional Figure 4**.

Detection of anti-enadenotucirev (EnAd) antibodies in patients following treatment with enadenotucirev.


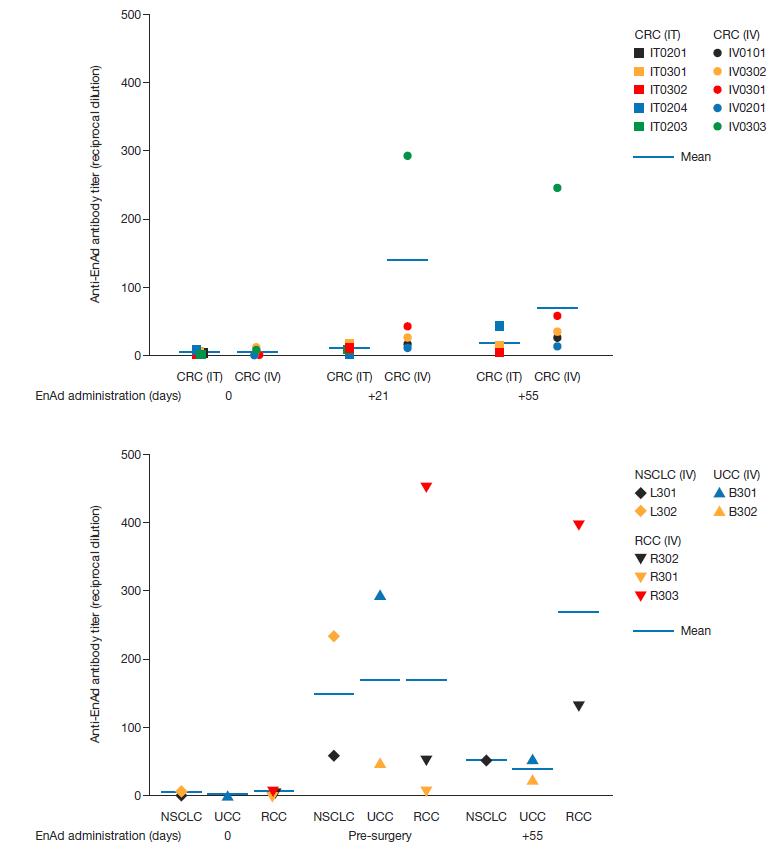


All samples were diluted 1:100 prior to analysis (to avoid serum inhibition), titer shown is not corrected for this pre-dilution.
