# Supplementary material for: Phase 1 study of intravenous administration of the chimeric adenovirus enadenotucirev in patients undergoing primary tumor resection
Source: J Immunother Cancer. 2017 Sep 19;5:71. doi: 10.1186/s40425-017-0277-7 (PMC5604344; doi:10.1186/s40425-017-0277-7)
Supplement: Supplementary file 2 — Table S1. Patient characteristics. Table S2. Nuclear staining of enadenotucirev in non-tumor tissue samples from patients with CRC (IT injection vs IV infusion). Table S3. Expression of the DNA mismatch repair proteins MLH1 and MSH2 in tumor samples from patients with CRC (IT injection and IV infusion). Table S4. Viral shedding in patients with CRC treated with enadenotucirev by IT injection (cohort A). (DOCX 80 kb) [file 40425_2017_277_MOESM2_ESM.docx]

Additional Table 1. Patient characteristics

| Cohort | Enadenotucirev delivery | Tumor type | *n* | Tumor  grade^a^ | Age  (median, years) | Male/female | BMI  (mean, kg/m^2^) |
| --- | --- | --- | --- | --- | --- | --- | --- |
| A | IT | CRC | 5 | G1:4  G2:1  G3:0 | 70.0 | 3/2 | 27.8 |
| B | IV | CRC | 5 | G1:1  G2:2  G3:2 | 78.0 | 3/2 | 30.5 |
| C | IV | NSCLC | 2 | G1:1  G2:1  G3:0 | 65.5 | 1/1 | 27.1 |
| D | IV | UCC | 2 | G1:0  G2:0  G3:2 | 69.0 | 2/0 | 32.6 |
| E | IV | RCC | 3 | G1:1  G2:1  G3:1 | 69.0 | 2/1 | 30.4 |

^a^Histopathological grade (G).

Additional Table 2. Nuclear staining of enadenotucirev in non-tumor tissue samples from patients with CRC (IT injection vs IV infusion)

| **Cohort** | **Patient** | **Normal tissue** | **Lymph node tissue** |
| --- | --- | --- | --- |
| A(IT enadenotucirev) | IT0201 | No staining | No staining |
|  | IT0203 | No staining one block; partial nuclear staining one block | Partial nuclear staining two blocks |
|  | IT0301 | No staining |  |
|  | IT0204 | Weak nuclear staining two blocks | Nuclear staining two blocks |
|  | IT0302 | No staining |  |
| B (IV enadenotucirev) | IV0101 | No staining |  |
|  | IV0201 | No staining | No staining |
|  | IV0301 | Weak nuclear staining |  |
|  | IV0302 | Weak nuclear staining |  |
|  | IV0303 | Weak nuclear staining |  |

Any staining seen in normal tissue was generally weaker than in tumor tissue.

Additional Table 3. Expression of the DNA mismatch repair proteins MLH1 and MSH2 in tumor samples from patients with CRC (IT injection and IV infusion)

| **Cohort** | **Patient** | **MLH1 IHC** | **MSH2 IHC** | **MSI status** |
| --- | --- | --- | --- | --- |
| A (IT enadenotucirev) | IT0201 | + | + | − |
|  | IT0203 | + | + | − |
|  | IT0204 | + | + | − |
|  | IT0301 | + | + | − |
|  | IT0302 | + | + | − |
| B (IV enadenotucirev) | IV0101 | + | + | − |
|  | IV0201 | + | + | − |
|  | IV0301 | + | + | − |
|  | IV0302 | + | + | − |
|  | IV0303 | + | + | − |

Additional Table 4. Viral shedding in patients with CRC treated with enadenotucirev by IT injection (cohort A)

|  |  | **Urine** | | **Saliva (buccal swab)** | | **Feces (rectal swab)** | |
| --- | --- | --- | --- | --- | --- | --- | --- |
| **Visit /**  **timepoint** | ***n*** | **Enadenotucirev concentration, vp/μL** | | | | | |
|  |  | **Mean** | **SD** | **Mean** | **SD** | **Mean** | **SD** |
| Day 1 | 5 | 0 | 0 | 0 | 0 | 68 | 62 |
| Day 2 | 5 | 0 | 0 | 0 | 0 | 206 | 209 |
| Day 4 | 4 | 0 | 0 | 0 | 0 | 1042^b^ | 1859 |
| Day 8 | 5 | 0 | 0 | 23^a^ | 0 | 169 | 196 |
| Day 15 | 4 | 0 | 0 | 0 | 0 | 57 | 65 |
| Day 22 | 5 | 0 | 0 | 23^a^ | 0 | 68 | 62 |
| Day 56 | 5 | 0 | 0 | 0 | 0 | 45 | 62 |

^a^One patient (IT0201) had 120 vp/μL at day 8 and one patient (IT0301) had 120 vp/μL at day 22.

^b^The maximum shed virus was 7.66 × 10^5^ vp/swab for Patient IT0201 on day 4, which decreased to 1.02 × 10^5^ vp/swab by day 8 and was below the quantifiable range at the next sampling point on day 15. The maximum shed virus for Patient IT0204 was 1.16 × 10^5^ vp/swab on day 2 and was below the quantifiable range at the next sampling point.

For results where viral DNA is present but less than the limit of quantitation of the assay (< 225 vp/μL), a value of 113 vp/μL was used for statistical analysis. Where no viral DNA was present in the sample, a value of 0 vp/μL was used for analysis. Enadenotucirev DNA concentration was summarized using descriptive statistics.
